# Comprehensive investigation of the clinical significance and molecular mechanisms of plasmacytoma variant translocation 1 in sarcoma using genome-wide RNA sequencing data

**DOI:** 10.7150/jca.31675

**Published:** 2019-08-27

**Authors:** Jianwei Liu, Rong Li, Xiwen Liao, Bangli Hu, Jia Yu

**Affiliations:** 1 Department of Spine Surgery, The Third Affiliated Hospital of Guangxi Medical University, Nanning, 530031, Guangxi Zhuang Autonomous Region, People's Republic of China.; 2 Department of Reproductive Center, The Third Affiliated Hospital of Guangxi Medical University, Nanning, 530031, Guangxi Zhuang Autonomous Region, People's Republic of China.; 3 Department of Hepatobiliary Surgery, The First Affiliated Hospital of Guangxi Medical University, Nanning, 530021, Guangxi Zhuang Autonomous Region, People's Republic of China.; 4 Department of Research, Affiliated Tumor Hospital of Guangxi Medical University, Nanning, 530021, Guangxi Zhuang Autonomous Region, People's Republic of China.

**Keywords:** sarcoma, plasmacytoma variant translocation 1, RNA sequencing, The Cancer Genome Atlas, molecular mechanism.

## Abstract

**Objective**: The present study aims to identify the potential clinical application and molecular mechanism of plasmacytoma variant translocation 1 (*PVT1*) in patients with sarcomas by mining an RNA sequencing dataset from The Cancer Genome Atlas (TCGA) through multiple genome-wide analysis approaches.

**Methods**: A genome-wide RNA sequencing dataset was downloaded from TCGA, survival analysis was used to evaluate the prognostic value of *PVT1* in sarcoma. The potential mechanism was investigated by multiple tools: Database for Annotation, Visualization, and Integrated Discovery v6.8, gene set enrichment analysis (GSEA), and Connectivity Map (CMap).

**Results**: Comprehensive survival analysis indicated that overexpression of *PVT1* was significantly associated with poor prognosis in patients with sarcoma, and nomogram demonstrated that *PVT1* contributed more than other traditional clinical parameters in sarcoma survival prediction. Weighted gene co-expression network analysis identified ten hub differentially expressed genes (DEGs) between sarcoma tissues with low and overexpression of *PVT1*, and substantiated that these DEGs have a complex co-expression network relationship. CMap analysis has identified that antipyrine, ondansetron, and econazole may be candidate targeted drugs for sarcoma patients with *PVT1* overexpression. GSEA revealed that overexpression of *PVT1* may be involved in the posttranscriptional regulation of gene expression, tumor invasiveness and metastasis, osteoblast differentiation and development, apoptosis, nuclear factor kappa B, Wnt, and apoptotic related signaling pathways.

**Conclusions**: Our findings indicate that *PVT1* may serve as a prognostic indicator in patients with sarcoma. Its underlying mechanism is revealed by GSEA, and CMap offers three candidate drugs for the individualized targeted therapy of sarcoma patients with overexpression of *PVT1*.

## Introduction

Malignant tumors derived from mesenchymal tissue (including connective tissue and muscle) are called "sarcomas" and occur mostly in the skin, under the skin, periosteum and both ends of the long bones. Sarcoma is a rare heterogeneous tumor, mainly originating from the embryonic mesoderm. Sarcoma has significant histological diversity, and the common histology types are osteosarcoma, leiomyoma, lymphosarcoma, and synovial sarcoma 1. At present, the main treatment for most sarcomas is surgical resection, patients whose tumors cannot be removed or have residual tumors following surgery are treated with radiotherapy 2. However, the blood transfer of sarcoma can occur in early stages of the disease, therefore, more than half sarcoma patients present with a high-risk of metastasis and death 1. The traditional prognostic indicators are histologic grade, the tumor size and depth, and the presence of distant or nodal metastases. However, these indicators cannot provide accurate target therapy for sarcoma. As with most tumors, the sarcoma is caused by the interactions of the gene and environment. Genetic analysis of sarcomas can provide targets for therapy and identify genetic markers. Therefore, the exploration of diagnostic and prognostic genetic indicators of sarcoma is crucial for guiding clinical treatment.

The Cancer Genome Atlas (TCGA) uses large-scale sequencing-based genomic analysis to understand the molecular mechanisms of cancer, raise awareness of the molecular basis of cancer, and improve our ability to diagnose, treat, and prevent cancer. TCGA contains 33 types of cancer multi-omics sequencing data, including sarcomas 3, 4. Plasmacytoma variant translocation 1 (*PVT1*) has been reported as an oncogene which is up-regulated in tumor tissues in various cancers and the overexpression of *PVT1* is correlated to a poor clinical outcome 5, 6. However, a comprehensive investigation of the clinical values of *PVT1* in sarcoma has not been reported. The present study aims to identify the potential clinical application and molecular mechanism of *PVT1* in patients with sarcomas by mining an RNA sequencing (RNA-seq) dataset from TCGA and by using multiple genome-wide analysis approaches.

## Materials and Methods

### Data source and pre-processing

An RNA-seq dataset of sarcoma tumor tissues was downloaded from TCGA (https://tcga-data.nci.nih.gov/; accessed November 1, 2018) 7, and the corresponding clinical and prognostic parameters were obtained from the University of California Santa Cruz Xena (UCSC Xena; http://xena.ucsc.edu/; accessed November 1, 2018). The raw count data of the RNA-seq was normalized by the *DESeq* package in R platform 8. The sarcoma patients with complete outcome data and RNA-seq data were included in the subsequent analysis. All data used in the current study were collected from the public database, therefore, additional approval by an ethics committee was not necessary. All data acquired and used in this study are in accordance with the publication guidelines of TCGA (https://cancergenome.nih.gov/publications/publicationguidelines).

### Survival analysis of* PVT1*

Survival analyses were conducted on patients with normalized mRNA expression and overall survival (OS) profiles. Patients were divided into low expression and overexpression groups according to the* PVT1* upper quarter expression value. Multivariate Cox proportional hazards regression analysis was applied to estimate the prognostic value of* PVT1* in sarcoma. A time-dependent receiver operating characteristic (ROC) curve was constructed using the *survivalROC* package in the R platform 9. Stratified analysis was used to evaluate the prognostic value of *PVT1* in different subgroups of sarcoma patients. The nomogram consisting of *PVT1* expression level and clinical parameters was used to individualize the prognosis evaluation of sarcoma patients.

### Identification of differentially expressed genes (DEGs) and functional assessment

To discover the mechanisms of prognosis for different *PVT1* expression levels, DEG screening was performed using the *edgeR* package in the R platform 10. A gene with a |log_2_fold change (FC)|>2 and false discovery rate (FDR)<0.05 was considered as DEG between the low and overexpression *PVT1* groups. Functional assessment of these DEGs was identified by using the Database for Annotation, Visualization and Integrated Discovery version 6.8 (DAVID v6.8, https://david.ncifcrf.gov/home.jsp; accessed November 1, 2018) 11, 12.

### Weighted gene co-expression network analysis (WGCNA) and hub DEGs screening

In order to screen for hub DEGs, we used a WGCNA method to construct a co-expression network 13, and the number of degrees in the networks was used to identify hub DEGs. A detailed introduction to WGCNA can be found in our previous publication 14. The soft threshold power selection was in accordance with the standard scale-free distribution, and the threshold of weighted co-expression correlation coefficient between two genes greater than 0.6 was included in the hub DEG screening and weighted co-expression network construction. The co-expression relationship was also verified by GeneMANIA (http://www.genemania.org/; accessed November 1, 2018)^15^, ^16^ and Search Tool for the Retrieval of Interacting Genes/Proteins (STRING, https://string-db.org/; accessed November 1, 2018)^17^, ^18^.

### Connectivity Map (CMap) analysis

CMap (https://portals.broadinstitute.org/cmap/; accessed November 1, 2018) is a biological application database of small-molecule drugs, gene expression profiles, and diseases, which is based on the differential gene expression of human cells treated with small-molecule drugs 19, 20. At present, more than 1,309 drug molecules and 7,000 gene expression profiles have been collected and shared on the CMap website. Each small-molecule drug was treated at different concentrations, different cell lines, and different time points 19, 20. Gene expression profile datasets were divided into positive and negative regulatory gene clusters to calculate the similarity of the gene atlas and then output the connective score. A positive connective score indicates the small-molecule drugs have an inductive effect on the query gene set, whereas a negative connective score indicates that the small-molecule drugs have an inhibiting effect on the query gene set. In this study, small-molecule drugs with a mean connective score of < -0.2 were regarded as possible therapy drugs for sarcoma patients with *PVT1* overexpression. The chemical structures of these compounds were downloaded from PubChem (https://pubchem.ncbi.nlm.nih.gov/; accessed November 1, 2018)^21^, ^22^ and protein-chemical interaction networks were investigated by Search Tool for Interacting Chemicals (STITCH: http://stitch.embl.de/; accessed November 1, 2018) 23-25.

### Genome-wide *PVT1* co-expression networks construction

It is well known that lncRNAs do not encode proteins and play a role mainly through regulating specific co-expressed protein-coding genes (PCGs). Therefore, we performed a genome-wide co-expression analysis of *PVT1* using the *cor* function in the R platform to assess the function of *PVT1* in sarcoma using the Pearson correlation coefficient. When the |Pearson correlation coefficient| between *PVT1* and other genes was greater than 0.4 and *P*-value <0.05 were identified as *PVT1* co-expression PCGs. DAVID v6.8 was then used in the functional enrichment of these *PVT1* co-expression genes.

### Gene set enrichment analysis (GSEA)

The underlining mechanisms of *PVT1* in sarcoma was also investigated by GSEA (http://software.broadinstitute.org/gsea/index.jsp; accessed November 1, 2018), which is a computational method that determines whether an a priori defined set of genes shows statistically significant concordant differences between two biological states (e.g. phenotypes)^26^, ^27^. The a priori defined gene sets included in the present study were downloaded from the Molecular Signatures Database (MSigDB), we only investigated the c2 (c2.all.v6.2.symbols.gmt) and c5 (c5.all.v6.2.symbols.gmt) gene sets in the current study 28. The significant results of GSEA were identified using the criteria: nominal *P*-value <0.05 and FDR <0.25.

### Statistical analysis

FDR in the GSEA and* edgeR* were adjusted for multiple testing with the Benjamini-Hochberg procedure 29-31. Survival analysis was performed by using the Kaplan-Meier method with the log-rank test, and Cox proportional hazards regression model with hazard ratios (HRs) and 95% confidence intervals (CIs). All statistical analyses were conducted with SPSS version 22.0 (IBM Corporation, Armonk, NY, USA) and R3.3.1 (https://www.r-project.org/). *P*-value <0.05 was considered statistically significant.

## Results

### Clinical features

There were 257 sarcoma patients included in the present study. We observed that patients with sarcoma with an age of more than 65 years, residual tumor (R1/R2/Rx), and multinodular were significantly associated with a poor OS (**Table 1**). In the multivariate Cox proportional hazards regression model, we also observed that patients with an age of more than 65 years (adjusted P=0.018, adjusted HR=2.099, CI=1.138-3.872,** Table 1**) and residual tumors (R1/R2/Rx, adjusted P=0.013, adjusted HR=2.146, CI=1.176-3.914,** Table 1**) may be an independent prognostic indicator for sarcoma OS and increased risk of death.

### Prognostic value of *PVT1* in sarcoma

**Figure 1:**
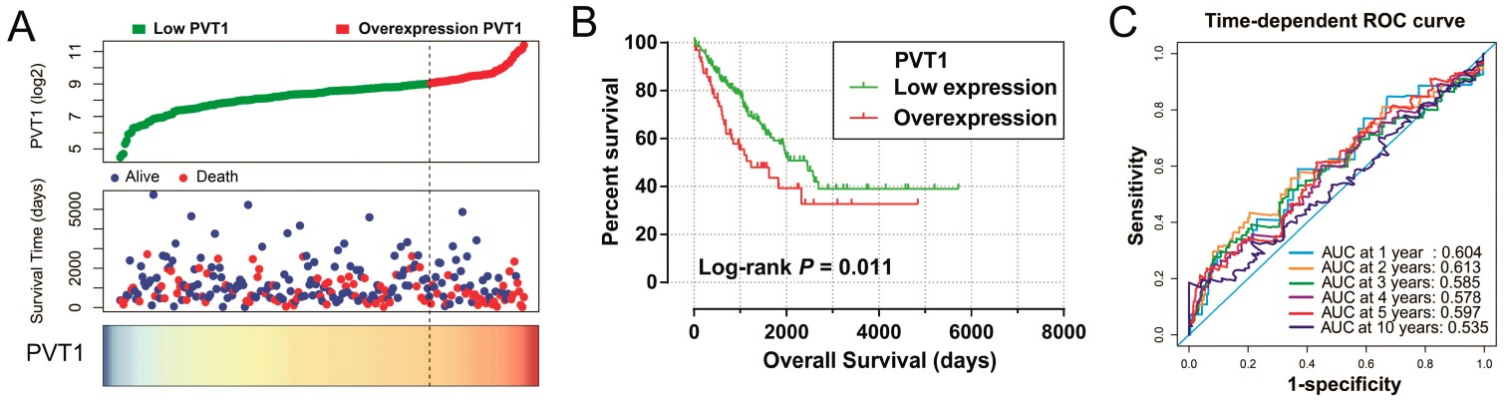
Prognostic value of *PVT1* in patients with sarcoma. **Notes**: (A) From top to bottom are the distribution plot of *PVT1* expression and patients' survival status and heat map of *PVT1* expression. (B) Kaplan-Meier curves for the low and overexpression *PVT1* groups. (C) ROC curves for predicting OS in sarcoma patients by* PVT1* expression. **Abbreviation:** *PVT1*, plasmacytoma variant translocation 1; AUC, area under the curve; ROC, receiver operating characteristic.

**Figure 2:**
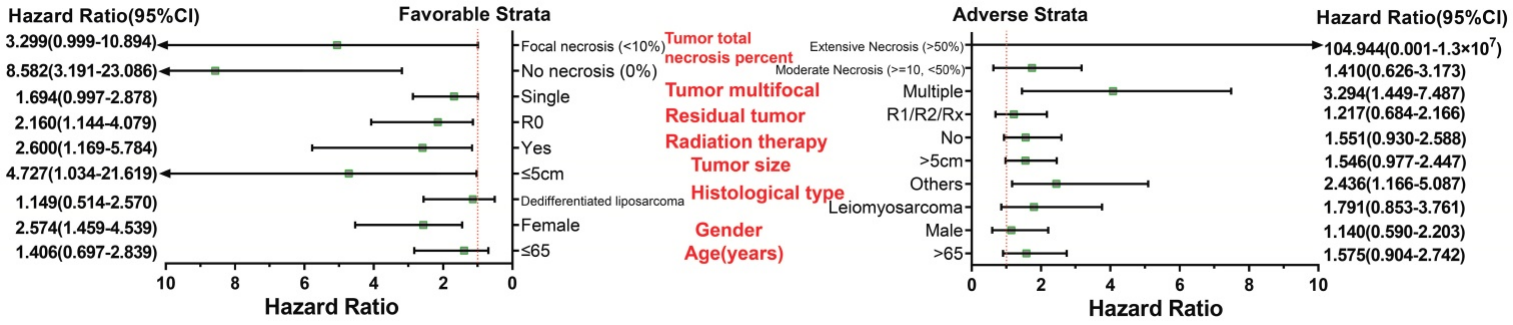
Stratified analysis of *PVT1* expression in sarcoma OS. **Abbreviation:** *PVT1*, plasmacytoma variant translocation 1; OS, overall survival.

**Figure 3:**
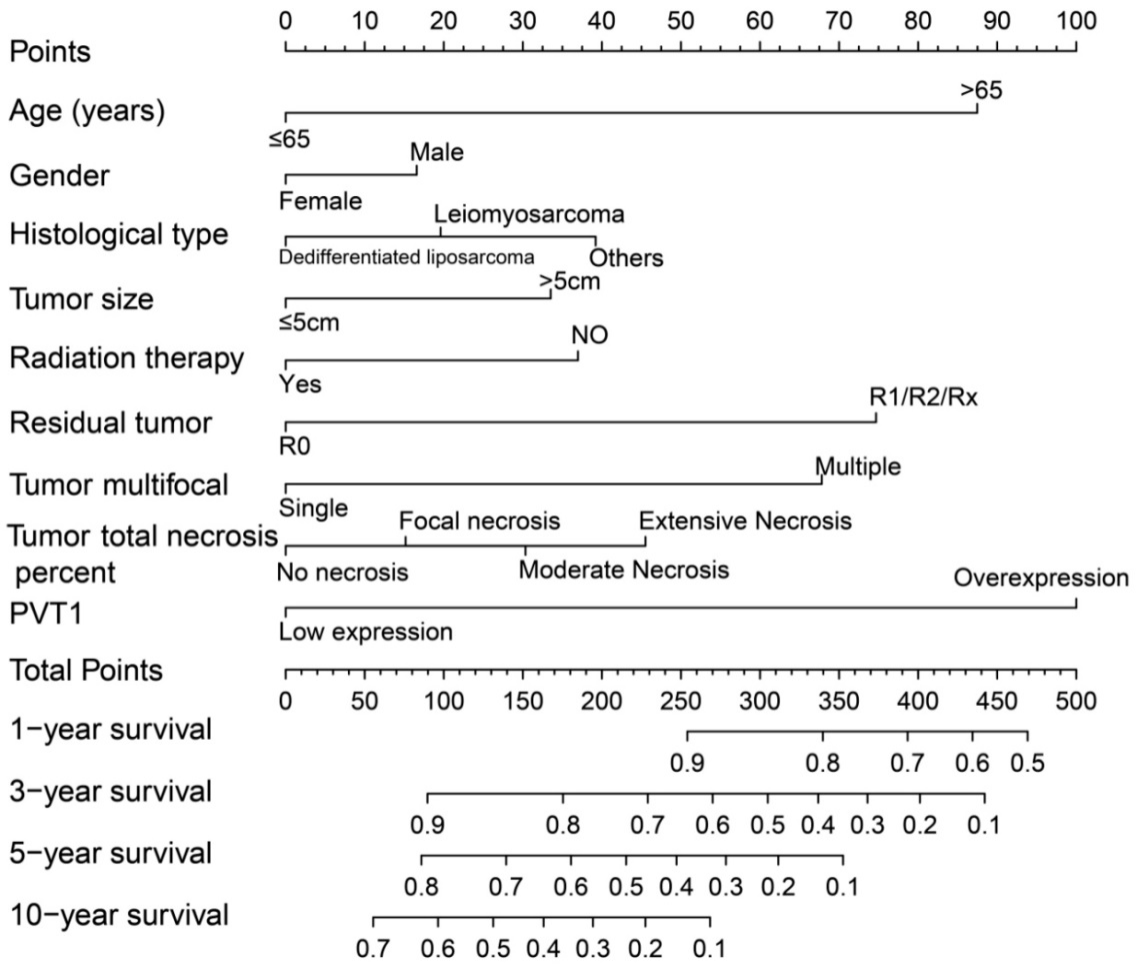
Nomogram of *PVT1* and other clinical information for sarcoma 1-, 2-, 3-, 4-, 5- and 10-year event (death) prediction. **Abbreviation:** *PVT1*, plasmacytoma variant translocation 1.

Survival analysis indicated that an overexpression of *PVT1* was significantly correlated to an unfavourable sarcoma OS (**Figure 1A, B**), and multivariate Cox proportional hazards regression model substantiated *PVT1* may be an independent prognostic indicator for sarcoma OS and increased risk of death (adjusted P=0.003, adjusted HR=2.590, CI=1.393-4.816,** Table 1**). Time-dependent ROC showed that *PVT1* expression can predict the OS at 1 and 2 years for sarcoma patients, with an area under the curve (AUC) of 0.604 and 0.613 (**Figure 1C**), respectively. Furthermore, we also performed a stratified analysis to assess the prognostic value of *PVT1* in different subgroups, and observed that *PVT1* was significantly associated with poor OS in patients with subgroups of residual tumor (R0), radiation therapy, no necrosis (0%), tumor size ≤5 cm, multinodular, female, and others histological type (**Figure 2**). The nomogram of *PVT1* and others clinical parameters showed that *PVT1* will contribute to the most points in the model, compared to other traditional clinical parameters (**Figure 3**).

### DEG screening and functional assessment

**Figure 4:**
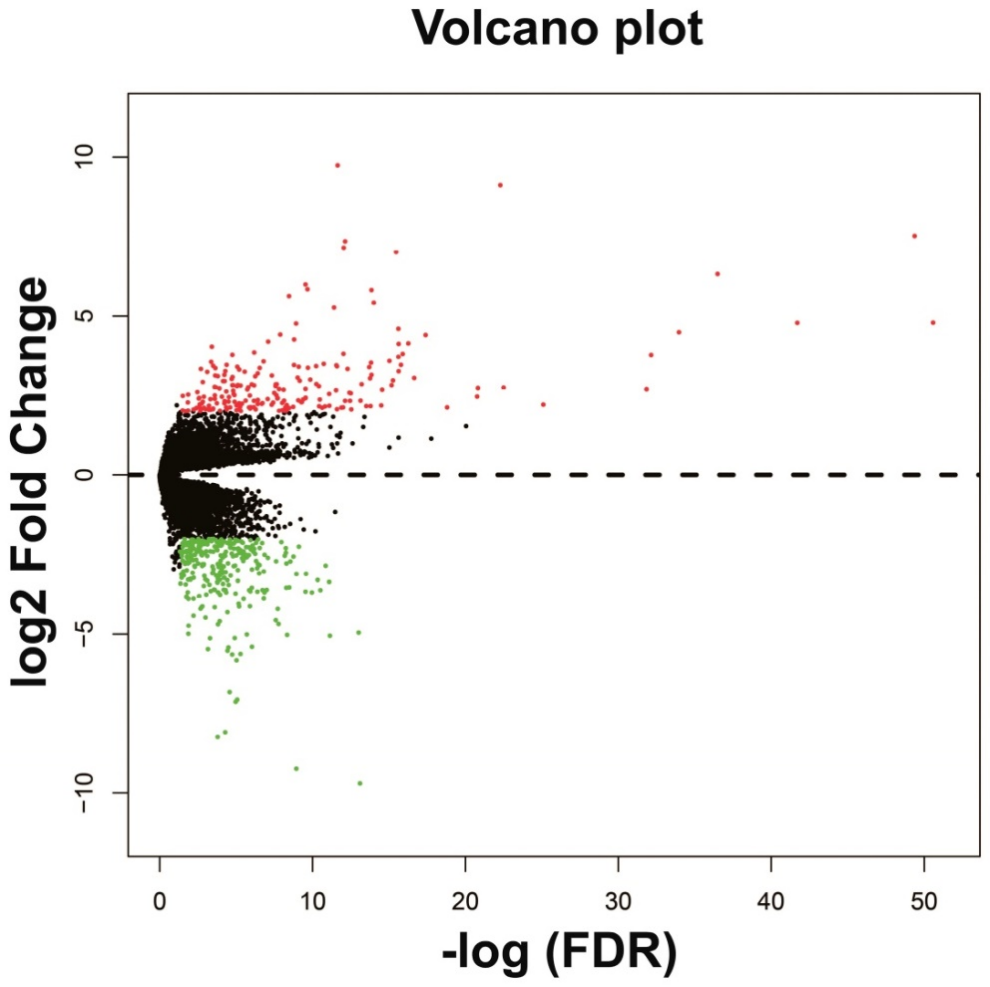
Volcano plots of differentially expressed genes between low and overexpression *PVT1* groups. **Abbreviation:** *PVT1*, plasmacytoma variant translocation 1; FDR, false discovery rate.

The DEGs between low expression and overexpression *PVT1* were identified by edgeR, and a total of 517 genes were identified as DEGs. Of these 517 genes, 306 DEGs were down-regulated and 211 DEGs were up-regulated when *PVT1* was overexpressed in tumor tissues (**Figure 4 and Figure S1**). Functional enrichment of gene ontology (GO) suggests that these DEGs were significantly enriched in multiple biological processes, such as sarcomere organization, stem cell differentiation, cell differentiation, and cell surface receptor signaling pathway (**Table S1**). Whereas the Kyoto Encyclopedia of Genes and Genomes (KEGG) enrichment suggest that these DEGs were significantly enriched in the calcium signaling pathway and tight junction.

### WGCNA and hub DEG screening

**Figure 5:**
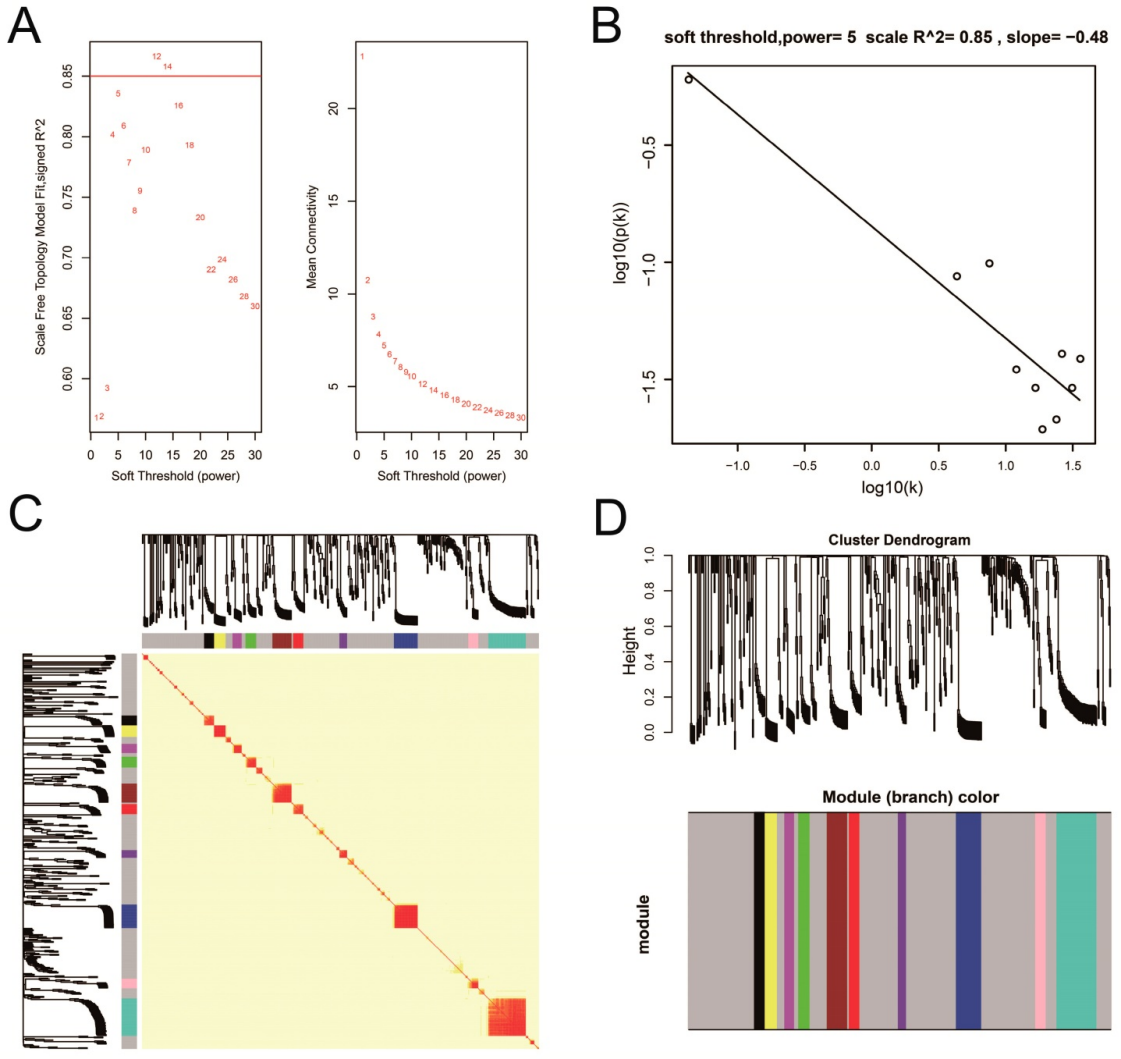
Plot of WGCNA analysis. **Notes:** (A) Soft threshold screening plot; (B) Scale-free topology plot; (C) Clustering dendrograms of genes; (D) TOM plot. **Abbreviation:** WGCNA**,** weighted gene co-expression network analysis; TOM, topological overlap matrix.

**Figure 6:**
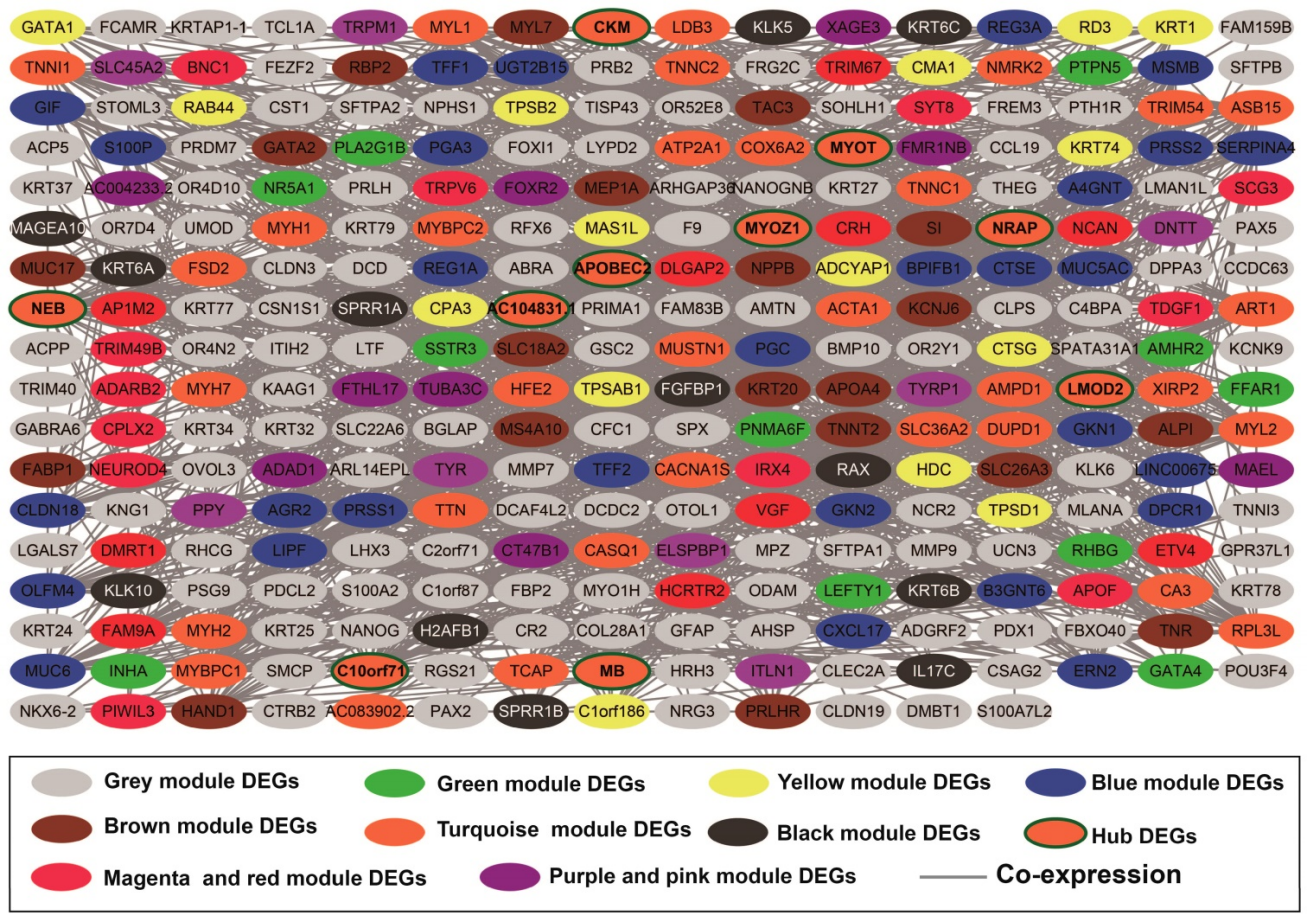
Weighted co-expression network for DEGs between low expression and overexpression *PVT1* groups. **Abbreviation:** DEGs, differentially expressed genes.

Co-expression networks of DEGs were constructed using the WGCNA method and validated by GeneMANIA and STRING. The soft threshold power in the present study was set as 5 (**Figure 5A, B**), these DEGs have been divided into 11 modules by module analysis (**Figure 5C, D, Table S3**). The DEGs in the gray module representation do not belong to any cluster. The WGCNA co-expression network is shown in **Figure 6 and Table S4**, and we identified that the top ten-degree DEGs were identified as hub DEGs. The hub DEGs in the WGCNA co-expression network of sarcoma tumor tissues were as follows: chromosome 10 open reading frame 71 (*C10orf71*), myoglobin (*MB*), nebulin related anchoring protein (*NRAP*), *AC104831.1,* apolipoprotein B mRNA editing enzyme catalytic subunit 2 (*APOBEC2*), creatine kinase, M-type (*CKM*), leiomodin 2 (*LMOD2*), myotilin (*MYOT),* myozenin 1 (*MYOZ1*), and nebulin (*NEB*).

**Figure 7:**
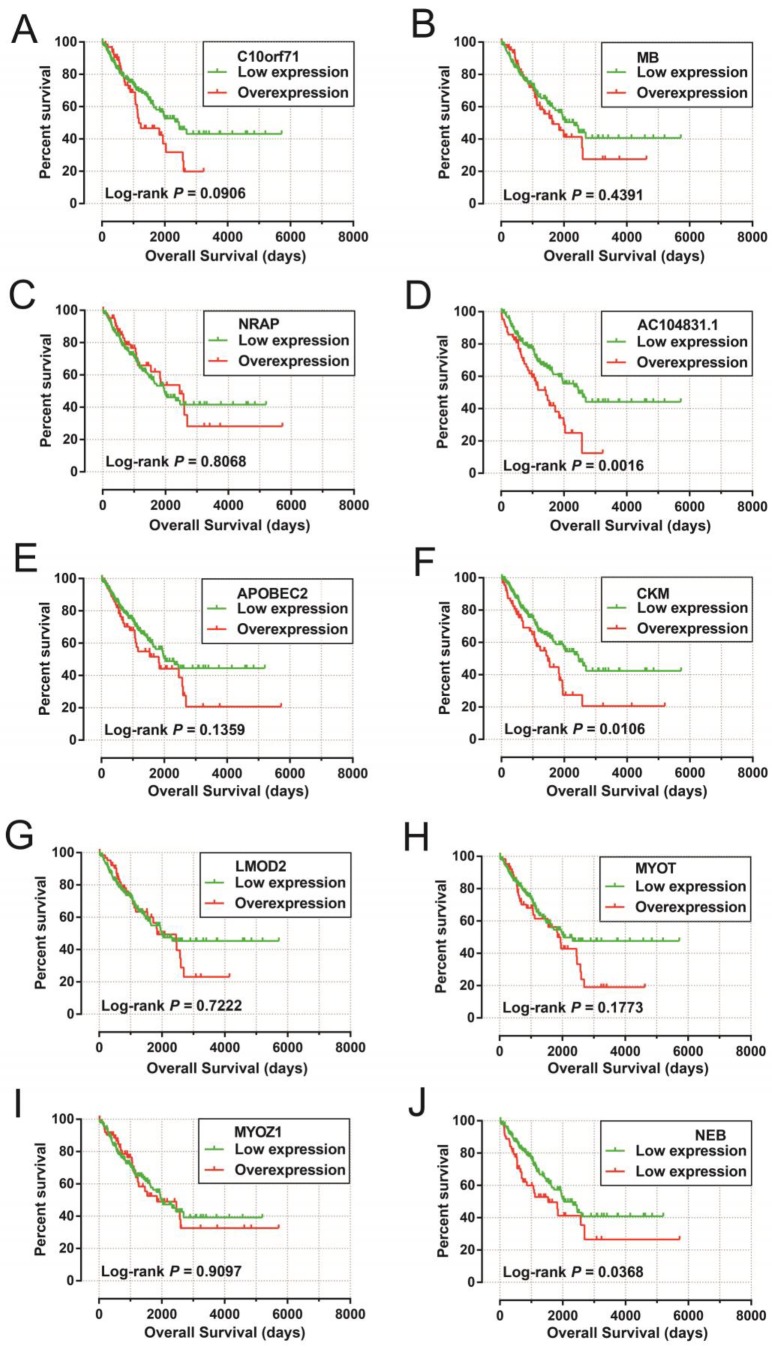
Kaplan-Meier survival curves of hub DEGs in weighted co-expression networks. **Notes**: Overall survival stratified by* C10orf71* (A), *MB* (B), *NRAP* (C),* AC104831.1*(D)*, APOBEC2* (E),*CKM* (F), *LMOD2* (G), *MYOT* (H)*, MYOZ1* (I) and *NEB* (J). **Abbreviation:** *C10orf71,* chromosome 10 open reading frame 71;*MB,* myoglobin; *NRAP,* nebulin related anchoring protein;* APOBEC2,* apolipoprotein B mRNA editing enzyme catalytic subunit 2; *CKM,* creatine kinase, M-type; *LMOD2,* leiomodin 2; *MYOT,* myotilin; *MYOZ1,* myozenin 1; *NEB,* nebulin.

**Figure 8:**
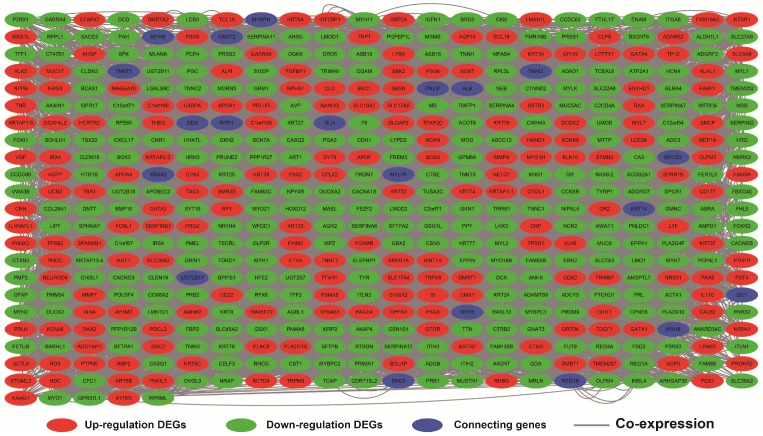
Co-expression network of DEGs constructed by GeneMANIA. **Abbreviation:** DEGs, differentially expressed genes.

**Figure 9:**
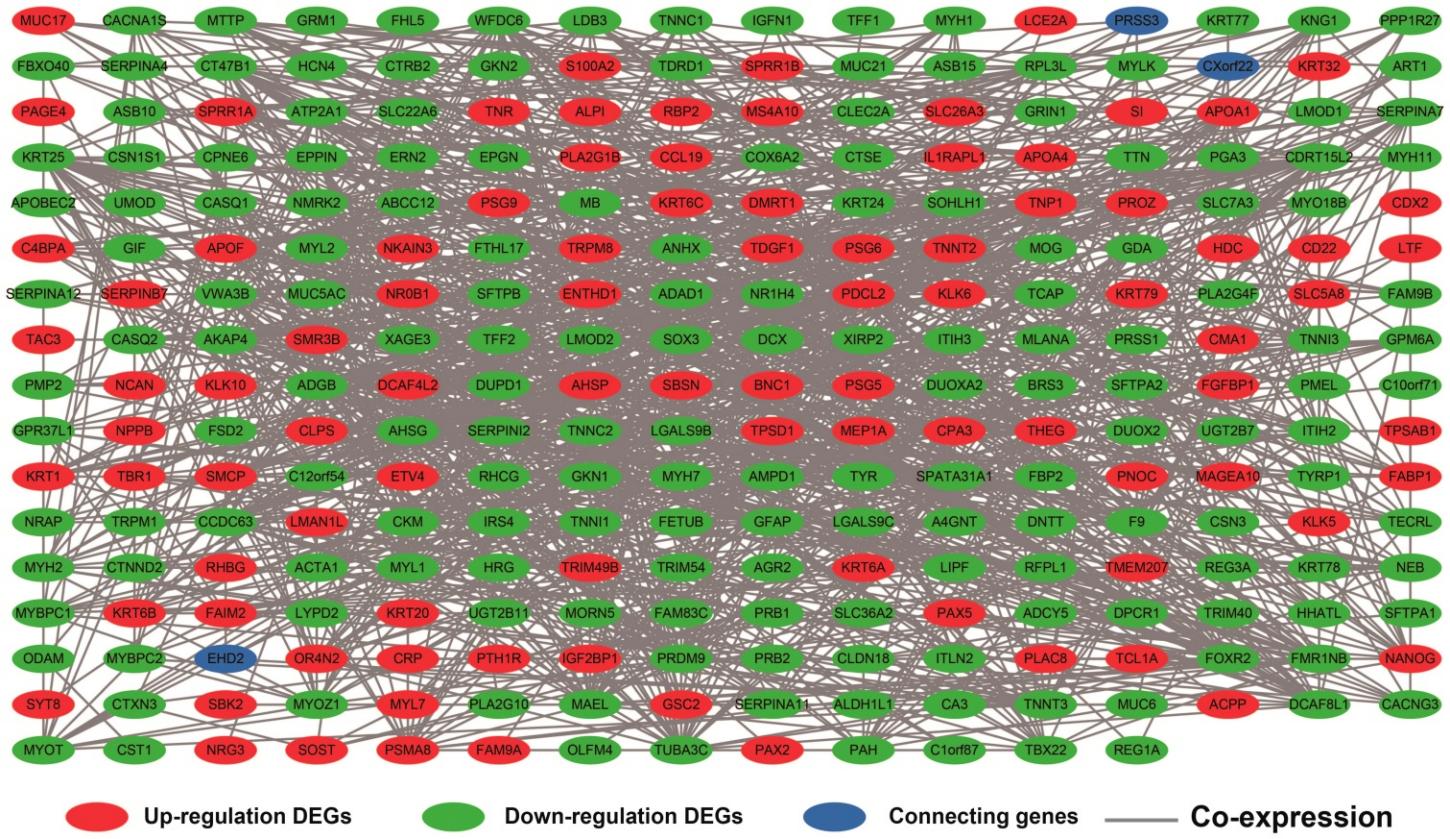
Co-expression network of DEGs constructed by STRING. **Abbreviation:** DEGs, differentially expressed genes.

The DEGs with the highest degrees in the network were C10orf71, MB and NRAP, with 39 degrees. Survival analysis (**Figure 7A-J**) of the top ten hub DEGs indicate that overexpression of *AC104831.1, CKM*, and *NEB* was significantly associated with poor OS in patients with sarcoma. To verify the co-expression relationship of these DEGs, we also performed a GeneMANIA (**Figure 8**) and STRING (**Figure 9**) network interaction analysis and observed that these DEGs have a complex relationship of co-expression and interaction, respectively.

### CMap Analysis

**Figure 10:**
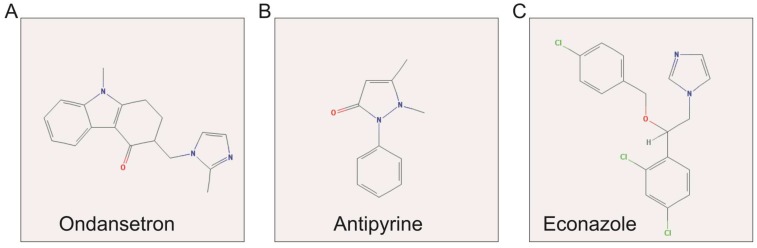
Structures of three candidate small-molecule drugs. **Notes**: (A) ondansetron; (B) antipyrine; and (C) econazole.

**Figure 11:**
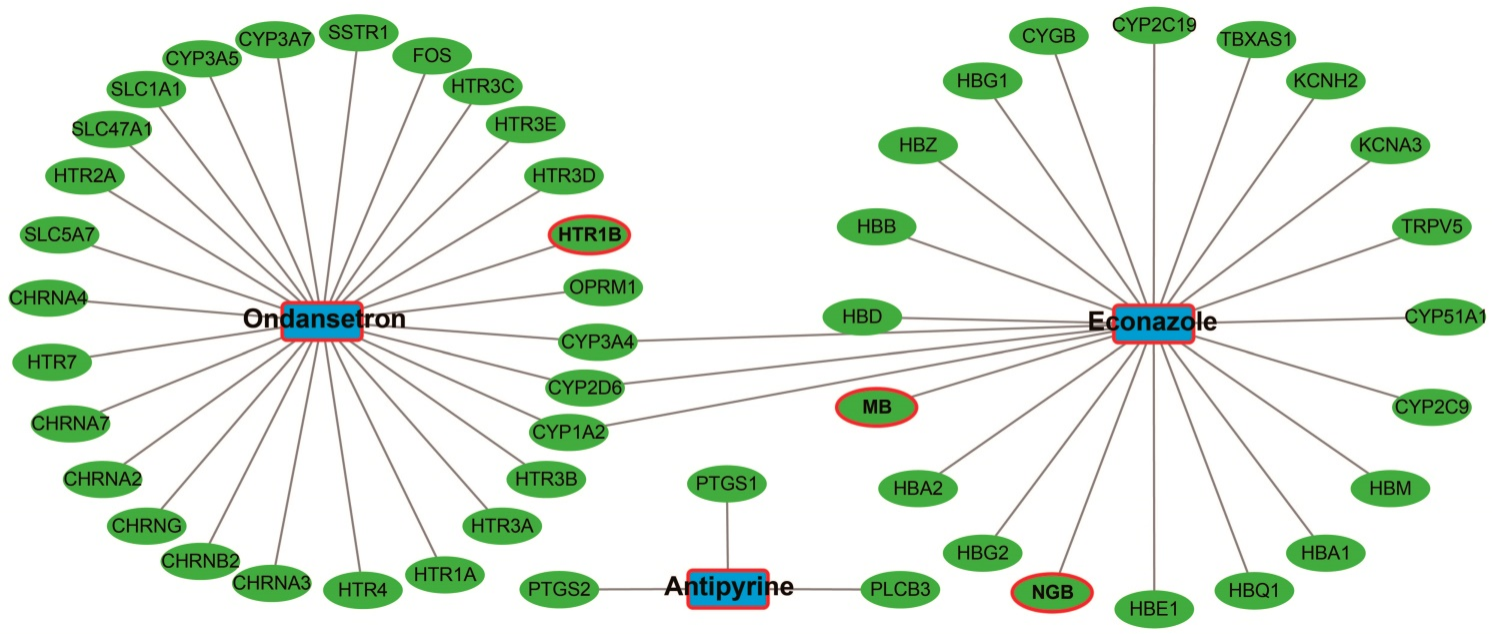
Candidate small-molecule drug-target genes interaction network.

The up-regulated and down-regulated DEGs were input into CMap to query. Only three small-molecule drugs with a mean connective score <-0.2 were identified as potential targeted drugs for sarcoma patients with overexpression of *PVT1*. The chemical structures of these three small-molecule drugs are shown in **Figure 10,** they are antipyrine, ondansetron, and econazole (**Table 2**). The protein-chemical interaction networks are shown in** Figure 11**. In the protein-chemical interaction networks, we observed three DEGs between *PVT1* low and overexpression groups. They are neuroglobin (*NGB*), *MB*, and 5-hydroxytryptamine receptor 1B (*HTR1B*). Therefore, we conclude that ondansetron may have an inhibiting effect on *PVT1* in sarcoma by regulating *HTR1B*, whereas, econazole inhibition was through regulating *NGB* and *MB*. In addition, because *MB* is a hub DEG in the WGCNA network of sarcoma tumor tissues, we conclude that econazole plays a role in its suppression mainly through the regulation of *MB*. However, further experiments are needed to confirm this hypothesis.

### Genome-wide *PVT1* co-expression PCG network construction

**Figure 12:**
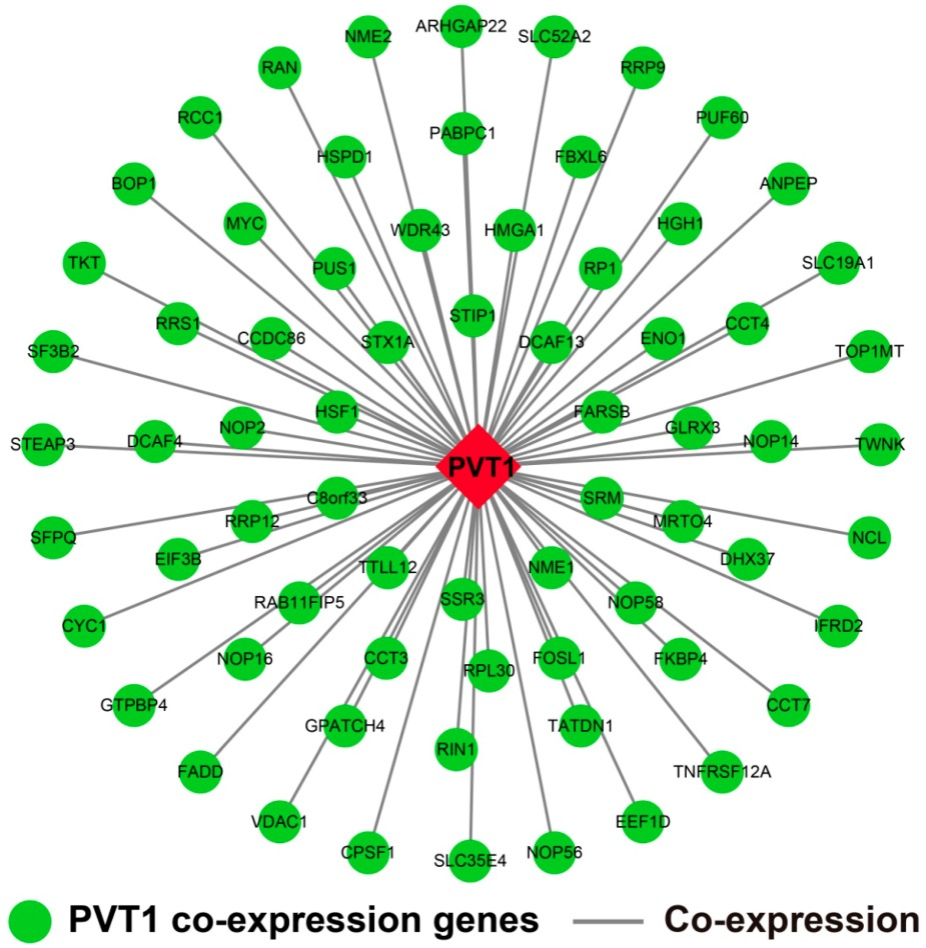
*PVT1* co-expression networks in sarcoma tumor tissues. **Abbreviation:*** PVT1*, plasmacytoma variant translocation 1.

The genome-wide *PVT1* co-expression PCG network construction was performed with 18,143 PCGs from 257 sarcoma tumor tissues. A total of 69 genes with the |Pearson correlation coefficient| >0.4 and *P*-value <0.05 were identified as *PVT1* co-expression PCGs (**Figure 12**). Functional assessment of these co-expression PCGs was carried out by using DAVID v6.8. GO term enrichment suggests that these co-expression PCGs were significantly correlated with mRNA transcription, angiogenesis, heat shock protein binding, RNA binding, cyclin-dependent protein kinase activating kinase holoenzyme complex, positive regulation of epithelial cell proliferation, and cell-cell adhesion (**Table S5**). The KEGG analysis suggests that these co-expression PCGs were involved in ribosome biogenesis in eukaryotes, RNA degradation, and biosynthesis of antibiotics (**Table S6**).

### GSEA

**Figure 13:**
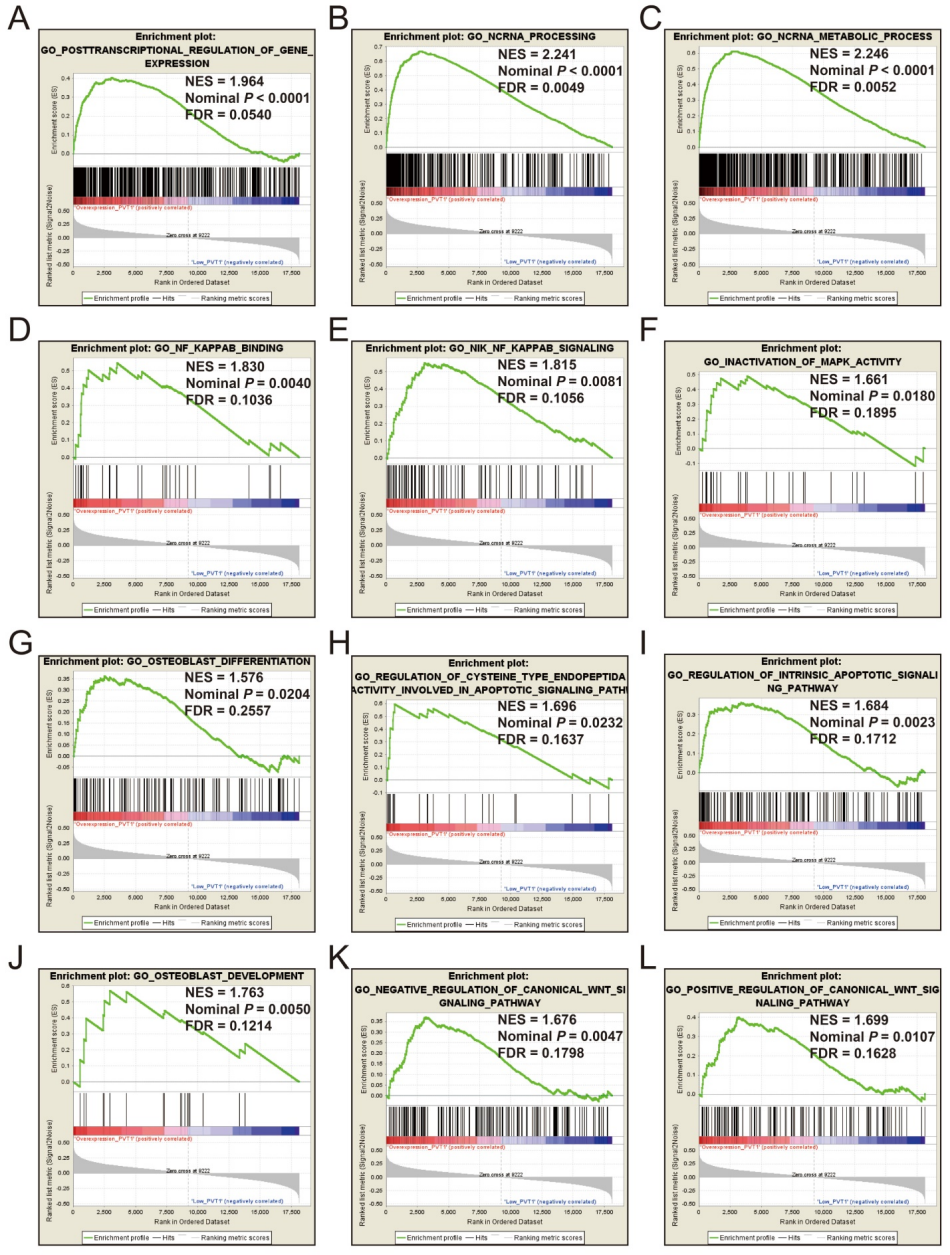
GSEA results of c5 reference gene sets for overexpression* PVT1* groups. (A) GO POSTTRANSCRIPTIONAL REGULATION OF GENE EXPRESSION, (B) GO NCRNA PROCESSING, (C) GO NCRNA METABOLIC PROCESS, (D) GO NF KAPPAB BINDING, (E) GO NIK NF KAPPAB SIGNALING, (F) GO INACTIVATION, (G) GO OSTEOBLAST DIFFERENTIATION, (H) GO REGULATION OF CYSTEINE TYPE ENDOPEPTIDA ACTIVITY INVOLVED IN APOPTOTIC SIGNALING PATHWAY, (I) GO REGULATION OF INTRINSIC APOPTOTIC SIGNALING PATHWAY, (J) GO OSTEOBLAST DEVELOPMENT, (K) GO NEGATIVE REGULATION OF CANONICAL WNT SIGNALING PATHWAY, (L) GO POSITIVE REGULATION OF CANONICAL WNT SIGNALING PATHWAY. **Abbreviations**: ES, enrichment score; NES, normalized enrichment score; FDR, false discovery rate; GSEA, gene set enrichment analysis;* PVT1*, plasmacytoma variant translocation 1.

**Figure 14:**
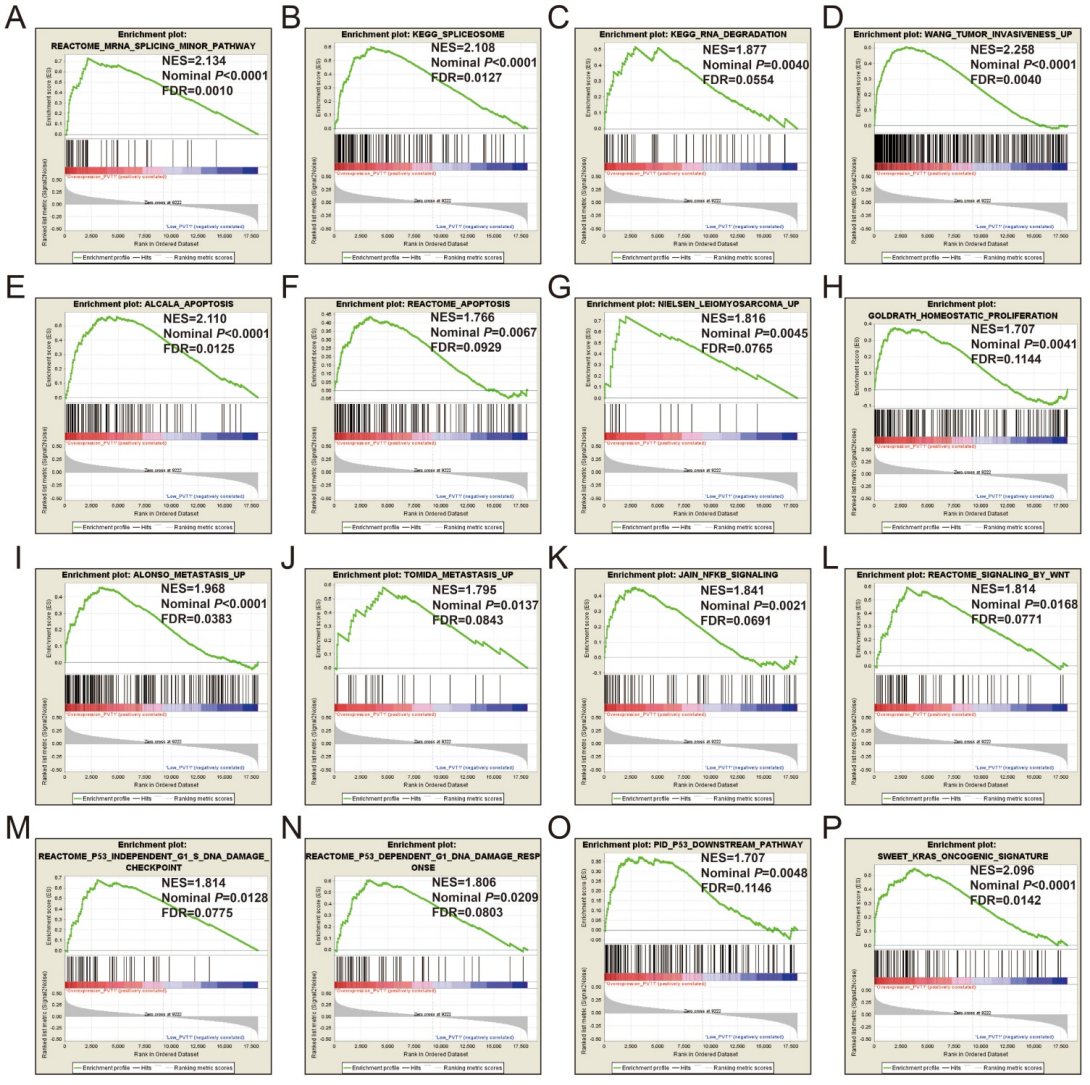
GSEA results of c2 reference gene sets for overexpression* PVT1* groups. (A) REACTOME MRNA SPLICING MINOR PATHWAY, (B) KEGG SPLICEOSOME, (C) KEGG RNA DEGRADATION, (D) WANG TUMOR INVASIVENESS, (E) ALCALA APOPTOSIS, (F) REACTOME APOPTOSIS, (G) NIELSEN LEIOMYOSARCOMA UP, (H) GOLDRATH HOMEOSTATIC PROLIFERATION, (I) ALONSO METASTASIS UP, (J) TOMIDA METASTASIS UP, (K) JAIN NFKB SIGNALING, (L) REACTOME SIGNALING BY WNT, (M) REACTOME P53 INDEPENDENT G1 S DNA DAMAGE CHECKPOINT, (N) REACTOME P53 DEPENDENT G1 DNA DAMAGE RESPONSE, (O) PID P53 DOWNSTREAM, (P) SWEET KRAS ONCOGENIC SIGNATURE. **Abbreviations**: ES, enrichment score; NES, normalized enrichment score; FDR, false discovery rate; GSEA, gene set enrichment analysis; *PVT1*, plasmacytoma variant translocation 1.

GSEA of c5 a priori defined gene sets indicate that the prognosis of *PVT1* overexpression is mainly involved in the difference of the following multiple biological processes: posttranscriptional regulation of gene expression, ncRNA processing, ncRNA metabolic process, nuclear factor kappa B (NF-KB) signaling pathway, inactivation of mitogen-activated protein kinase (MAPK) activity, osteoblast differentiation and development, regulation of apoptotic signaling pathway, and Wnt signaling pathway (**Figure 13A-L**, **Table S7**). The GSEA of a c2 a priori defined gene set indicates that mRNA splicing minor pathway, spliceosome, RNA degradation, tumor invasiveness, apoptosis, leiomyosarcoma, homeostatic proliferation, metastasis, NF-KB and Wnt signaling pathway, Kras oncogenic signature, and tumor protein p53 pathway (**Figure 14A-P**, **Table S8**) were significantly enriched in groups of *PVT1* was overexpressed.

## Discussion

*PVT1* has been reported to be up-regulated in multiple cancer tumor tissues and identified as an oncogene by functional experiments 32. Due to differential expression between tumor and normal tissues, it is also considered as a diagnostic biomarker for a variety of cancers 32-34. The up-regulation of *PVT1* in tumor tissues can be observed in 18 types of cancers in the TCGA database 32, moreover, several other cohort studies have confirmed this 5, 35-40. The oncogene role of *PVT1* in cancers have been identified in multiple cancers including bladder cancer 41, nasopharyngeal carcinoma (NPC) 40, colorectal cancers (CRC) 37, non-small-cell lung cancer (NSCLC) 42, 43, clear cell renal cell carcinoma (ccRCC) 36, gastric cancer (GC) 44-46, head and neck squamous cell carcinoma (HNSCC)^35^, prostate cancer (PC) 34, esophageal squamous cell carcinoma (ESCC) 47, osteosarcoma 48, 49, hepatocellular carcinoma (HCC)^50^, ^51^, and pancreatic ductal adenocarcinoma (PDAC) 52.

The prognostic values of *PVT1* were also investigated in multiple cancer types in multiple cohorts. Large-scale survival analysis of *PVT1* using TCGA database was performed by He and his co-workers and it was observed that *PVT1* expression is up-regulated in tumor tissues compared with paired non-cancer tissues and significantly correlates to a worse clinical outcome and advanced stage in human cancers 5.

In their investigation of the prognosis of sarcoma from a TCGA cohort, they did not observe that the expression of *PVT1* was significantly associated with sarcoma OS because their original dataset was extracted from the Oncolnc website (http://www.oncolnc.org/), which is a third-party data integration website of TCGA database, and the cut-off value of low- and high-expression groups were according to the median value 5. A similar study by Chen et al. also performed a large-scale survival analysis of *PVT1* by using the TCGA RNA-seq data from the UCSC Xena website (https://xenabrowser.net/heatmap/) and assessed this data by a univariate Cox proportional hazards model, they observed that low- and high-expression of *PVT1* grouped by the median values were significantly associated with sarcoma OS 53. Although the prognosis analysis of TCGA sarcoma *PVT1* was analyzed, both of these previous studies extracted the *PVT1* expression data from a third-party data integration website of TCGA. Due to the RNA-seq data processing methods, patient inclusion and exclusion criteria between the UCSC Xena and Oncolnc websites may be different, this will result in inconsistent results of the same cohort in these two previous studies, users cannot control the quality of the original data. In addition, these two previous studies did not make full use of the advantages of whole genome data to carry out comprehensive mining, but only stayed at the level of prognosis analysis, therefore, the potential expression profiling, functions, pathways, mechanism, and targeted therapeutic drugs of *PVT1* in sarcoma remain unclear. Thus, it is necessary to use genome-wide analysis tools to further explore the molecular mechanism of *PVT1* and screen potential targeted therapeutic drugs. In the present study, we grouped the low and overexpression of *PVT1* by the upper quarter expression value, which can more accurately reflect the high expression level of *PVT1*. The advantage of our study is that we made full use of the whole genome dataset to screen the DEGs between different *PVT1* expression levels, and identified hub DEGs by the WGCNA method. Through the DEGs, we can further perform an investigation to screen the potential small-molecule drugs for targeted therapy of *PVT1* overexpression by using the CMap online tools. Furthermore, we also used the genome-wide RNA-seq dataset to perform a GSEA between different *PVT1* expression groups and identified the potential mechanism of *PVT1* in sarcoma prognosis. Consistent with previous studies, we found that *PVT1* is a biomarker of a poor prognosis in sarcoma and can be a novel therapeutic target.

Extensive previous studies have observed that the high expression of *PVT1* significantly increased the risk of death in patients with GC 33, 44-46, 54, 55, CRC 37, 56, NSCLC 43, 57, ccRCC 36, 58, 59, uveal melanoma 60, HNSCC 35, 61, PDAC 52, NPC 40, PC 34, ESCC 47, ovarian cancer (OC) 62, high-grade serous carcinoma 63, cervical cancer 64, 65, and osteosarcoma 48, 49. Xu et al. also identified and verified that *PVT1* was significantly correlated to tumor metastasis of ccRCC by using a comprehensive genome-wide analysis 59. In terms of tumor progression, multiple previous studies have observed that *PVT1* is up-regulated in advanced stage tumor tissues, and significantly associated with malignant progression in patients with ESCC 47, OC 62, and HNSCC 61. Multiple meta-analysis studies have comprehensively evaluated these previous studies, and suggest that *PVT1* could serve as a novel biomarker for metastasis, clinical stage, and poor prognosis in various tumors 6, 53, 66.

For functional assessment of DEGs and *PVT1* co-expression genes, we found that these genes enriched in multiple biological processes and pathways were associated with oncogenic function. The biological processes of the regulation of stem cell differentiation, cell differentiation, cell surface receptor signaling pathway, cell-cell adhesion, and angiogenesis were correlated to tumorigenesis and progression 44, 46, 67. Through literature review indicated that the three small-molecule drugs for *PVT1* targeted therapy identified in the present study have not been reported to be associated with sarcoma treatment in previous studies. The effectiveness of these candidate small-molecule targeted drugs still needs further experimental verification.

For GSEA mechanism investigations, we have enriched multiple biological processes and pathways that were related to the characteristics of long non-coding RNA, including posttranscriptional regulation of gene expression, ncRNA processing, ncRNA metabolic process, and RNA degradation. Functional related mechanisms are enriched in apoptotic, Wnt, MAPK, and NF-KB signaling pathway, which were significantly associated with tumorigenesis. Previous studies have demonstrated that *PVT1* promotes angiogenesis by activating specific pathways in cancers 44, 67. Similar functional studies of *PVT1* can also be found in other types of cancers. *PVT1* up-regulation in CRC promotes tumor cell proliferation, invasion, and metastasis. The promotion of cancer metastasis by *PVT1* can also be observed in HCC 68, as well as its inductive effect on apoptosis in HCC 69. *PVT1* may participate in the induction of apoptosis by regulating p53 70, and play an oncogenic role by regulating mutant p53 35.

Multiple previous studies have also substantiated the relationship of *PVT1* as an oncogene involved in Wnt and NF-KB signaling pathways 51, 61, 71. Most of these GSEA enrichment results of *PVT1* in sarcoma were consistent with previous studies, and we infer that *PVT1* can be a novel therapeutic target for sarcoma and may have clinical application values in sarcoma prognosis monitoring and the designation of treatment strategy.

There were several limitations that need to be explained clearly in the present study. Our findings were obtained only from TCGA sarcoma patient cohorts with a sample size of 257, a future study with a large sample size to verify our results is needed. Second, because the sample size in the present study was small, we did not observe that *PVT1* showed a good prognostic predictive ability in all subgroups of sarcoma. Third, due to the absence of some clinical parameters of patients with sarcoma in TCGA, we failed to include all prognostic factors of sarcoma in the survival analysis and nomogram construction. Despite the limitations mentioned above, our study is the first time to comprehensively investigate the clinical significance and prospective molecular regulatory mechanism of *PVT1* in sarcoma using a whole genome RNA-seq dataset, discover its clinical significance in predicting the prognosis of sarcoma, and identify three potential *PVT1* targeted drugs using CMap. Once the above findings are confirmed, *PVT1* will play an important role in the treatment and prognosis monitoring of sarcoma.

## Conclusion

Our findings indicate that *PVT1* can serve as a prognosis biomarker in patients with sarcoma, and performed well in short-term OS prediction. By screening the DEGs between different *PVT1* expression groups, we have identified ten hub DEGs, as well as DEG co-expression network identification and validation. Furthermore, we also identified three potential small-molecule drugs (antipyrine, ondansetron, and econazole) for targeted therapy of *PVT1* overexpression in sarcoma patients. The potential molecular mechanism of *PVT1* in sarcoma may involve the posttranscriptional regulation of gene expression, tumor invasiveness and metastasis, osteoblast differentiation and development, apoptosis, NF-KB, Wnt, and apoptotic related signaling pathways. However, our results still need further confirmation by experiments *in vivo* and* in vitro* and in clinical trials.

## Supplementary Material

Supplementary figure.

Supplementary tables.

## Figures and Tables

**Table 1:** Univariate and multivariate survival analysis of *PVT1* and clinical parameters of the sarcoma cohort in TCGA.

Variables	Events/total (n=257)	MST (days)	Univariate analysis			Multivariate analysis	
			HR (95% CI)	*P*		HR (95% CI)	*P*
**Age (years)**							
≤65	46/157	2599	1			1	
>65	52/100	1164	2.362(1.584-3.523)	<0.0001		2.099(1.138-3.872)	0.018
**Gender**							
Female	57/140	1991	1			1	
Male	41/117	1970	0.874(0.585-1.306)	0.511		1.177(0.664-2.087)	0.576
**Histological type**							
Dedifferentiated liposarcoma	25/58		1			1	
Leiomyosarcoma (LMS)	41/104		0.842(0.512-1.385)	0.498		1.464(0.539-3.977)	0.454
Others §	32/95		0.823(0.478-1.389)	0.466		1.521(0.600-3.854)	0.377
**Tumor size £**							
≤5cm	8/37	2599	1			1	
>5cm	84/209	1941	1.727(0.836-3.570)	0.14		1.301(0.496-3.417)	0.593
**Radiation therapy ₤**							
Yes	27/74	1953	1			1	
NO	70/177	1991	1.155(0.740-1.801)	0.526		1.330(0.724-2.442)	0.358
**Residual tumor &**							
R0	44/153	NA	1			1	
R1/R2/Rx	54/103	1175	2.533(1.697-3.781)	<0.0001		2.146(1.176-3.914)	0.013
**Tumor multifocal Ψ**							
Single	64/188	2448	1			1	
Multiple	26/49	1941	1.680(1.065-2.652)	0.026		1.935(0.954-3.926)	0.067
**Tumor total necrosis percent**							
No necrosis(0%)	20/69	2575	1			1	
Focal necrosis(<10%)	14/38	2448	1.427(0.720-2.830)	0.309		1.833(0.850-3.951)	0.122
Moderate Necrosis(≥10,<50%)	26/61	1536	1.609(0.896-2.888)	0.111		1.377(0.675-2.809)	0.379
Extensive Necrosis(>50%)	5/12	1941	1.409(0.527-3.771)	0.494		1.280(0.403-4.063)	0.675
***PVT1* expression level**							
Low expression	67/193	2448	1			1	
Overexpression	31/64	1235	1.727(1.127-2.647)	0.012		2.590(1.393-4.816)	0.003

Notes:§Others group including Myxofibrosarcoma; Giant cell MFH / Undifferentiated pleomorphic sarcoma with giant cells; Sarcoma, synovial, poorly differentiated; Synovial Sarcoma - Biphasic; Synovial Sarcoma - Monophasic; Undifferentiated Pleomorphic Sarcoma (UPS); Malignant Peripheral Nerve Sheath Tumors (MPNST). £ Information of tumor size was unavailable in 11 patients; ₤ Information of radiation therapy was unavailable in 6 patients; &Information of residual tumor was unavailable in 1 patients; Ψ Information of tumor multifocal was unavailable in 20 patients. **Abbreviation:** *PVT1*, plasmacytoma variant translocation 1; TCGA, The Cancer Genome Atlas; MST, median survival time; HR, hazard ratio; CI, confidence interval.

**Table 2:** Potential small-molecule drugs for sarcoma patients with overexpression of *PVT1* identified by CMap.

CMap name	mean connective score	n	enrichment	*P* value	specificity	percent non-null
**ondansetron**	-0.434	4	-0.81	0.00255	0.0075	50
**phenazone**	-0.461	3	-0.77	0.02498	0.0578	66
**econazole**	-0.374	4	-0.659	0.0306	0.1111	50

**Abbreviation:** *PVT1*, plasmacytoma variant translocation 1; CMap, Connectivity Map.
