# Supplementary material for: Comprehensive investigation of the clinical significance and molecular mechanisms of plasmacytoma variant translocation 1 in sarcoma using genome-wide RNA sequencing data
Source: J Cancer. 2019 Aug 27;10(20):4961–77. doi: 10.7150/jca.31675 (PMC6775530; doi:10.7150/jca.31675)
Supplement: Supplementary file 1 — Supplementary figure. [file jcav10p4961s1.pdf]

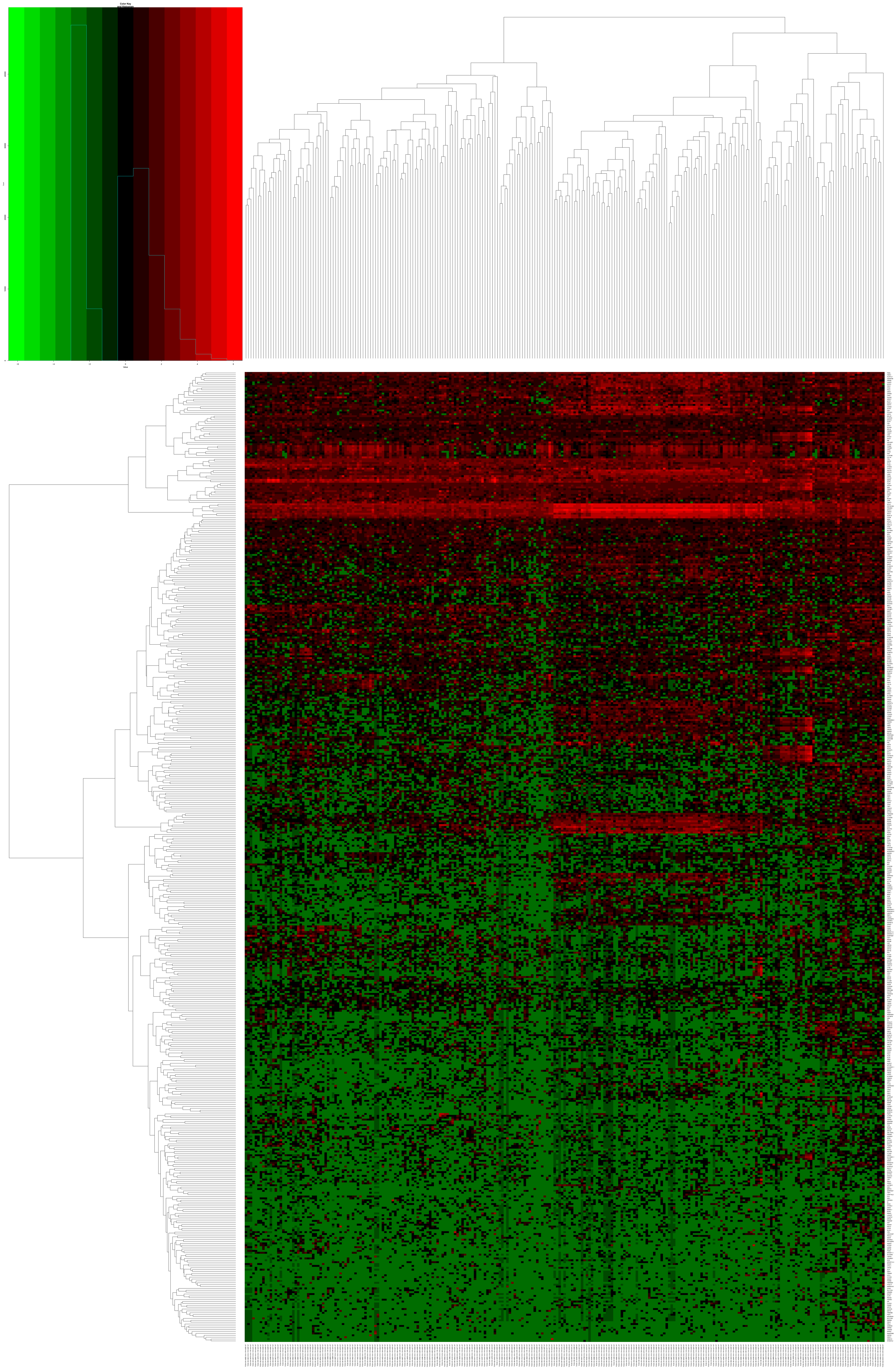

Figure S1. Heat map of DEGs between low and overexpression PVT1 groups.
